# A reconstruction algorithm for compressive quantum tomography using various measurement sets

**DOI:** 10.1038/srep38497

**Published:** 2016-12-14

**Authors:** Kai Zheng, Kezhi Li, Shuang Cong

**Affiliations:** 1 Department of Automation, University of Science and Technology of China, Hefei, 230027, China; 2 Imperial College London, London, W12 0NN, UK

## Abstract

Compressed sensing (CS) has been verified that it offers a significant performance improvement for large quantum systems comparing with the conventional quantum tomography approaches, because it reduces the number of measurements from *O*(*d*^2^) to *O*(*rd* log(*d*)) in particular for quantum states that are fairly pure. Yet few algorithms have been proposed for quantum state tomography using CS specifically, let alone basis analysis for various measurement sets in quantum CS. To fill this gap, in this paper an efficient and robust state reconstruction algorithm based on compressive sensing is developed. By leveraging the fixed point equation approach to avoid the matrix inverse operation, we propose a fixed-point alternating direction method algorithm for compressive quantum state estimation that can handle both normal errors and large outliers in the optimization process. In addition, properties of five practical measurement bases (including the Pauli basis) are analyzed in terms of their coherences and reconstruction performances, which provides theoretical instructions for the selection of measurement settings in the quantum state estimation. The numerical experiments show that the proposed algorithm has much less calculating time, higher reconstruction accuracy and is more robust to outlier noises than many existing state reconstruction algorithms.

In quantum information science, quantum state tomography (QST) is one of the essential tasks to many quantum mechanics problems, such as measuring gains of optical signals, and determining actual states of qubits in quantum computing^1^. Generally speaking, for an *n*-qubit quantum system, the state estimation process is equivalent to recovering a density matrix *ρ* of size *d* × *d* in a Hermitian space from *M* projected measurements, where the standard method needs *M*~*O*(*d*^2^), and *O*(*d*^4^) experimental configurations to fully characterize the state property^2^. Here each measurement is actually an estimate of the expectation value by averaging measurement outcomes corresponding to certain measurement basis. This large number of measurements makes measuring a quantum state with even a few qubits costly. As a result, efficient and robust methods that need fewer number of measurements in QST are desperately needed, as well as the related optimal measurement sets along with the algorithm.

To estimate the quantum state accurately with less colored effort, many estimation methods have been well studied. The most widely used ones are least-squares (LS)^3^, maximum-likelihood estimation (MSL)^4^,^5^,^6^ and some others^7^,^8^,^9^. In practice, quantum states in which people are interested are often in pure, or nearly pure, states such as ground states of a local Hamiltonian^10^, or states with low entropy^11^. In this case, the state or dynamics can be represented by low-rank or sparse process matrices approximately^12^,^13^,^14^,^15^, meaning the density matrix *ρ* is close to a matrix of rank *r* (due to local noise process), where *r*~*O*(1). Given this property as prior information, *O*(*rd*log*d*) measurement settings could possibly suffice in the implementation of tomography to reconstruct *ρ* thanks to the theory of compressed sensing^13^,^14^,^15^,^16^,^17^,^18^,^19^,^20^,^21^. By leveraging this technique, the number of measurements can be significantly reduced, and this has been verified successfully by real experiments^2^,^11^,^22^,^23^,^24^,^25^. In contrast to various methods for conventional QST though, there exist few algorithms proposed for CS based QST specifically^14^,^18^,^26^. Liu^18^ adopted the Dantzig algorithm to estimate the state. Li^26^ applied the Alternating Direction Method of Multipliers (ADMM) algorithm to quantum state estimation, and achieved a solution with good accuracy. Yet the high computational complexity of the methods limits their applications.

In this paper, we make two-fold contributions on reconstruction algorithm and measurement sets. We develop an improved algorithm specifically for compressive quantum state tomography to further accelerate the process of recovering *ρ*. Firstly the state estimation is converted to an optimization problem with quantum constraints. By leveraging the fixed point equation approach to avoid the matrix inverse operation, we propose a fixed-point alternating direction method of multipliers (FP-ADMM) algorithm for compressive quantum state estimation that can handle both normal errors and large outliers in the density matrix (for which LS and MSL can fail easily). Comparisons with other quantum estimation approaches in numerical experiment shows the advantage of the proposed method. In addition, besides the Pauli basis, other bases such as the Platonic Solid set and the Stokes set have also been claimed to be practical measurement sets in QST. Thus we analyze the performances of these three bases along with other two bases in the compressive QST model in terms of their matrix coherences/RIP constant, and establish a correspondence between the bound of bases and its reconstruction performance.

## Results

We have done a study in both using quantum state estimation via CS to reduce the number of measurement settings and fast optimization algorithm. 1) We analyzed five most popular measurement sets in quantum state estimation and adopt them to quantum state estimation via CS. The lower bound of the measurement rate was explored by numerical experiment. It shows that the required minimal measurement rate of the Pauli measurement set is less than the Platonic Solid measurement set and the Stokes measurement set, which makes the Pauli measurement set a preferred measurement set for quantum state estimation via CS. Though the Gaussian measurement set and Bernoulli measurement set’s required minimal measurement rate is even less, they are not implementable in practice to date. 2) Combining the fixed point equation and ADMM algorithm, we propose an algorithm for quantum state estimation via CS, the FP_ADMM algorithm. The FP_ADMM algorithm can greatly improve the time efficiency of the quantum state estimation (about 150 + faster for *n* = 7) and achieve a much higher reconstruction accuracy than ADMM algorithm (about 140 + %, *n* = 5, *η* = 0.2) and LS algorithm (about 420 + %, *n* = 5, *η* = 0.2).

### Quantum state estimation via CS Model

Quantum tomography or quantum state estimation is the process of reconstructing the density matrix *ρ* of size *d* × *d* for a source of quantum systems by measurements. Here, the measurements can be represented by a set of positive semidefinite matrices Π = {Π_*i*_, *i* ∈ Ω|Π ≥ 0, ∑_*i*_(Π_*i*_) = **1**}, where 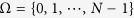, and an element of this set can be the possible outcome of one measurement. In the QST process, one measurement obtains one outcome, and the probability of obtaining outcome Π_*i*_ (or the probability of projecting *ρ* to the pure state 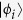) is given by the Born rule 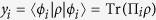, where 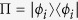 denotes a matrix projection operator in the equation. If we use 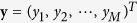 as the normalized probability vector of outcomes, 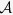 denotes the set of matrix projection operator Π, then 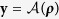. If the prior information is given that the rank of the density matrix is low, the quantum state estimation can be viewed as a process of CS reconstruction of solving a convex optimization problem^13^:


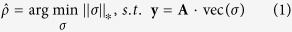


where || ⋅ ||_*_ is the nuclear norm, which equals to the sum of singular values, vec(⋅) represents the transformation from a matrix to a vector by stacking the matrix’s columns, and **A** is the matrix format of 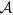. When the number of the outcomes of a measurement set is equal to the number of parameters to be estimated (which is *d*^2^ for a *d*-dimensional Hilbert space), meaning any two density matrices are distinguishable, the measurement set is known as informationally-complete; if it’s greater than the number of the parameters, the measurement set is referenced as over-complete.

In QST via CS, an incomplete measurement set is leveraged to reduce the number of measurement settings, which leads to a reduction of necessary number of the outcomes^14^. Considering the implement of quantum state estimation via CS, it is preferred to use a partial informationally-complete or partial over-complete measurement set


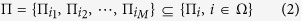


where *M* ≪ *N*, *N* ≥ *d*^2^, *M* is the number of outcomes. **A** is the matrix form of the incomplete measurement set of (2):


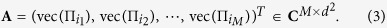


**A** reduces the dimension of values from *d* × *d* to *M* × 1. This is known as the measurement matrix or sampling operator. In quantum state estimation, every *i*th measurement is performed on a large number of identical copies. Quantum state estimation is an effective approach of using the outcomes of measurement set {***y***_*i*_, *i* ∈ Ω} and the measurement set Π_*i*_, *i* ∈ Ω to calculate the best-fit density matrix.Compressive

### Compressive Quantum State Estimation Algorithm using FP_ADMM

During the measuring process of quantum state estimation in practical application, noise exists due to the system or measurement errors. Normally the noise is assumed to satisfy certain distribution (like Gaussian). However there exist abnormal circumstances in the measuring process that may cause the perturbation in the density matrix, and of course the perturbation does not satisfy the Gaussian distribution. The perturbation can be reflected by sparse outlier entries in *ρ* and these outlier entries can be formulate as a sparse matrix *S*. Conversional QST algorithms (such as LS) can barely handle *S*, because the low-rank property will be significantly affected by these small portion of outliers. Taking *S* into consideration, optimization problem (1) becomes^26^:


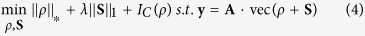


where *λ* is a compromise factor (*λ* > 0). 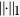 is the (1, 1) norm, 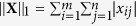. *I*_*C*_(*ρ*) denotes the indictor function on a convex set C, 
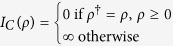
. The function of *I*_*C*_(*ρ*) is projecting *ρ* into a Hermitian matrix, for the reason that *ρ* has to satisfy *ρ* = *ρ*^†^, *ρ*^†^ represents the conjugate transpose of *ρ*. The optimization problem (4) can be solved by different estimators. In this paper, we use the estimator of matrix LASSO^27^, and the solution of (4) is the solution of the minimization of the augmented Lagrangian function:


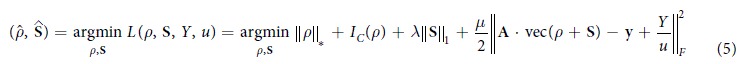


where *μ* > 0.

We find the solution of (5) by leveraging a so-called FP_ADMM algorithm we have proposed, which is based on ADMM and the fixed point equation^28^. The solution is represented as follows:


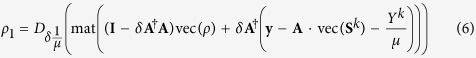



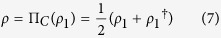



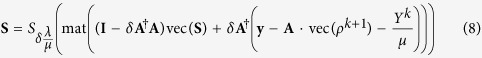


*S*_*λ*_(**X**) is the soft threshold defined as 
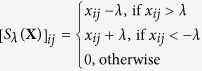
, *D*_*λ*_(**X**) is the singular value contraction operator defined as *D*_*λ*_(**X**) = *US*_*λ*_(*S*)*V*^*T*^, where *USV*^*T*^ is the singular value decomposition of **X**. The FP_ADMM solutions can be calculated in a recursive way (see the Methods section). The FP_ADMM algorithm can search for the global optimal solution for (6) and (8), and avoid computing the pseudo inverse of an extremely large matrix compared to ADMM. Hence, the FP_ADMM can improve the algorithm’s efficiency and reduce the computational complexity significantly.

### Choice of Measurement sets and Reconstruction Quality

The choice of measurement set is very important, because if the measurement set is not chosen properly, the density matrix can never be reconstructed accurately. There are several informationally-complete measurement sets which can provide excellent performance for quantum state estimation. An informationally-complete measurement set of an *n*-qubit system can be represented as:


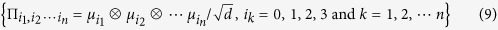


where *μ*_0_, *μ*_1_, *μ*_2_ and *μ*_3_ are four projection operators for a single qubit system. One of the most popular measurement sets was proposed by D. F. V. James^29^ in 2001, and we refer this measurement set as Stokes measurements. For the Stokes measurement set, 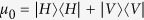, 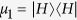, 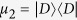, 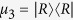. 
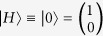
 is horizontal polarization, 
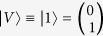
 is vertical polarization, 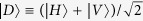 is diagonal polarization, and 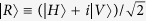 is right-circular polarization. One of the most used measurement set is Pauli measurements^13^,^30^. For the Pauli measurement set, *μ*_0_ = *I* is a two order unit matrix, and *μ*_1_ = *σ*_*x*_, *μ*_2_ = *σ*_*y*_, *μ*_3_ = *σ*_*z*_ are the Pauli matrices. Another measurement set proposed recently is Platonic solid measurements^31^. In this paper, we use tetrahedron Platonic solid measurement set whose 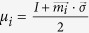, and 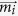 is a real three dimensional unit Bloch vector which matches the centers of the faces of a tetrahedron. 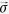 is a vector of the Pauli matrices (*σ*_*x*_, *σ*_*y*_, *σ*_*z*_).

In this paper, the incomplete measurement set (2) is a random subset of the informationally-complete measurement set. The measurement rate is defined as


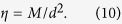


The smaller the *η* is, he smaller the number of measurement settings is. The reduction of *M* is the reduction of *η* for *η* is proportional to *M*. When *η* = 1, the measurement set is an informationally-complete measurement set. *η* is also called the compression ratio in CS. Liu^18^ proved that if the measurement rate of partial Pauli measurement set satisfies 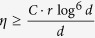, the Pauli satisfies rank RIP^32^ with very high probability. Here satisfying rank RIP implies the solution of the convex optimization problem (1) is unique and equal to the density matrix. It should be noted that satisfying rank RIP is a sufficient but not necessary condition for measurement matrix to guarantee the density matrix is reconstructed exactly. Even if a measurement matrix does not satisfies rank RIP, the density matrix may still be reconstructed accurately. We hope to explore the lower bound of the measurement rate. According to Dual Certification theory^33^, the Pauli measurement set is an ortho-normal set, and it can be calculated coherence *v* = 1. This implies that for Pauli measurement set, if *η* satisfies^33^


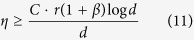


the solution of (1) is unique and equal to *ρ* with probability at least 1 − *d*^−*β*^ for any *β* > 0, where *C* is some absolute constant. This guarantees that when the number of measurement settings is *O*(*rd* log *d*), the measurement matrix constructed by the incomplete Pauli measurement set may not satisfy RIP according to Liu^18^, but it can still be used in quantum state estimation via CS and guarantee the density matrix be reconstructed exactly.

In CS, there are several types of measurement matrices that can reduce the required measurement rate dramatically. These matrices include matrices whose entries sampled from an i.i.d. symmetric Bernoulli distribution^32^, Gaussian measurement ensemble^34^ etc. The measurement matrix **A** in (3) of Bernoulli measurement can be written as: 
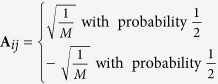
 and the form of Gaussian Measurement matrix is 
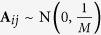
. It has been proved that about *O*(*rd* log *d*) measurement settings for Bernoulli measurement and about *O*(*rd*) measurement settings for Gaussian measurement can be used to reconstruct the density matrix exactly^32^,^34^. But these matrices have yet to be applied to the physical implement of quantum state estimation and will not be considered in this paper. As for the Stokes measurement set and Platonic Solid measurement set, neither of these measurement sets is ortho-normal^29^,^31^, so the dual certification theory cannot be applied. Whether these two partial measurement sets satisfy rank RIP has been unexplored in the literature. We will explore the lower bound of measurement rate of Stokes measurements and Platonic Solid measurements by numerical simulation(see the Numerical Simulation subsection).

To evaluate the reconstruction quality, the normalized error is defined as


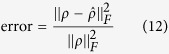


where *ρ* is the true state. We also notice that the trace norm distance is preferred in some quantum state estimation applications and the Frobenius norm distance is somehow more popular in compressive sensing application. As a matter of fact, Frobenius norm distance and trace norm distance are both suitable metrics in quantum state estimation as presented in refs 18,35 and the numerical simulation result shows that the normalized estimate error’s numerical value of Frobenius norm and trace norm are very close. As our research adopts compressive sensing, we decided to use Frobenius norm distance in this paper. In numerical simulations, *ρ* is generated from normalized Wishart random matrices with form as^36^:


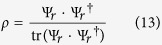


where Ψ_*r*_ is a complex *d* × *r* matrix with i.i.d. complex random Gaussian entries. The density matrix constructed by (13) satisfies the constraint that the trace of density matrix equals 1.

### Numerical Simulation

We carry out three experiments. The first experiment validates the Pauli measurement set’s lower bound of the measurement rate described in (11). The Platonic Solid measurement set and the Stokes measurement set’s lower bounds of measurement rate are also explored. Furthermore, we answer the question: in order to get a fixed estimation accuracy of 95% in practice, how many measurement settings exactly are required? The other two experiments demonstrate FP_ADMM’s superiority of accuracy and efficiency compared with LS and ADMM.

#### Exploration of the lower bound of measurement settings of five different measurement sets

**Figure 1:**
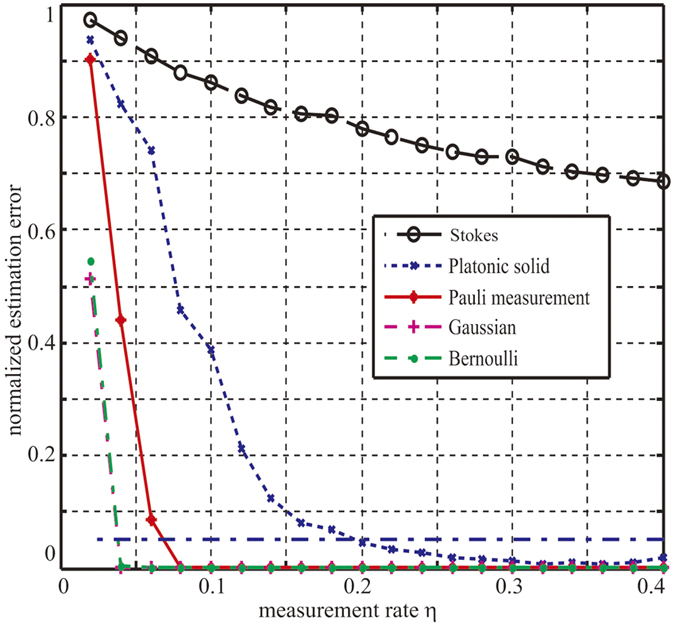
Normalized estimation error with different measurement rates under 5 measurement sets. The red star line, blue cross line, black circle line, magenta plus sign line and green dot line represent Pauli measurements, Tetrahedron Platonic Solid measurements, Stokes measurements, Gaussian measurement and Bernoulli measurement respectively. The blue dotted line represents the normalized estimation error is 0.05. If the normalized estimation error is greater than 1, we record it as 1. The measurement rate increases from *η* = 0.02 to *η* = 0.4, and the incremental step is Δ*η* = 0.02. Under each measurement rate, the algorithm runs the measurement and reconstruction 3 times and the estimation error is the mean value of the 3 normalized estimation errors, and the max number of iterations in every reconstruction is set as 100.

Figure 1 depicts the normalized estimation errors with different measurement rates under 5 different measurement sets. In this experiment, we recover the density matrix without outlier noise *S*, for the outlier does not affect the lower bound of measurement settings due to (11). The specific experimental method exploits the FP_ADMM algorithm at *n* = 6, to describe the relationship between the normalized estimation error and the measurement rate *η*. The measurement sets we choose are: 1) Pauli measurement set; 2) Tetrahedron Platonic Solid measurement set; 3) Stokes measurement set; 4) Gaussian measurement ensemble^34^; 5) Entries sampled from an i.i.d. symmetric Bernoulli distribution^32^.

#### Accuracy Comparison of FP_ADMM and ADMM and LS

**Figure 2:**
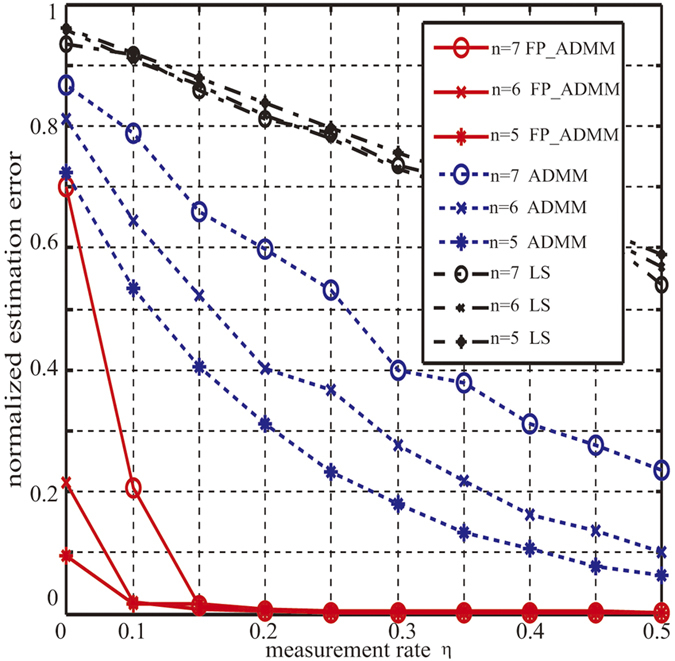
Comparison experimental results of FP_ADMM and ADMM and LS. The realization of ADMM and LS used the iterative method in ref. 26 and in ref. 3 respectively. The solid lines, dashed lines and dot dash lines represent the FP_ADMM and ADMM and LS respectively, and the circle lines represent *n* = 5, the cross lines represent *n* = 6, the star lines represent *n* = 7. The measurement rate increases from *η* = 0.05 to *η* = 0.5 and the incremental step is Δ*η* = 0.05. Under each measurement rate, the algorithms run the measurement and reconstruction 3 times, and the errors are the mean value of the 3 normalized estimation errors. In each reconstruction, the max number of iterations is 30.

To validate the superiority of FP_ADMM algorithm in terms of reconstruction accuracy, we use FP_ADMM, LS and ADMM to reconstruct the density matrix *ρ* by using the same measurement matrix **A** (Here we use the measurement matrix constructed by the Pauli measurement set) and the same outcomes **y**. Meanwhile, to validate the robustness of FP_ADMM, the outlier noise is added in the density matrix. The outlier noise **S** is setted with 0.01*d*^2^ nonzero values located uniformly randomly and the magnitudes satisfying the Gaussian distribution 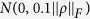. We record the normalized estimation errors of FP_ADMM and LS and ADMM under the different measurement rates at corresponding qubits *n* = 5, *n* = 6 and *n* = 7. The normalized estimation errors with increasing *η* and various qubits *n* = 5, *n* = 6, and *n* = 7 are shown in Fig. 2.

#### Efficiency Comparison between FP_ADMM and ADMM

In order to investigate the superiority of the FP_ADMM algorithm in the time efficiency of the quantum state estimation via CS, we record the single iteration time of the FP_ADMM algorithm and ADMM algorithm when the measurement rates are 0.1, 0.25, and 0.4 and *n* = 5, *n* = 6, and *n* = 7. The results of the FP_ADMM and ADMM under different measurement rates are shown in Table 1.

## Discussion

It can be observed from Fig. 1 that: 1) In the case of *n* = 6 for the Pauli measurement set and the Tetrahedron Platonic Solid measurement set, the measurement rate *η* needs to be at least 0.08 and 0.32 respectively to reconstruct the density matrix almost exactly. Here exact reconstruction implies error = 0. As for the Stokes measurement set, the incomplete measurement set cannot reconstruct the density matrix exactly. 2) The Partial Pauli measurement set, partial Tetra Platonic Solid measurement set, Gaussian measurement set and Bernoulli measurement set can reconstruct the density matrix exactly. However, the Gaussian measurement set and Bernoulli measurement set cannot be applied to the quantum estimation via CS in practice to date. To reconstruct the density matrix exactly, the required minimal measurement rate of the Pauli measurement set is less than the Platonic Solid measurement set, which makes the Pauli measurement set a preferred measurement set for quantum state estimation via CS. 3) According to (11), for the Pauli measurement set, when *n* = 6, the lower bound of measurement rate is 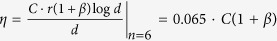 which equals 0.08 according to Fig. 1, implying that the factor *C*(1 + *β*) ≈ 1.23. Similarly, it can be calculated that *η* needs to be at least 0.13 to reconstruct the density matrix exactly for *n* = 5 and 0.05 for *n* = 7. 4) In practical application, the density matrix is usually considered to be reconstructed correctly when the normalized estimation error is very small. Here if estimation accuracy is 95% or more, that means *error* ≤ 0.05, and we take this as the density matrix being reconstructed correctly. It can be observed from Fig. 1 that: for FP_AADMM algorithm with 100 iterations, the density matrix can be reconstructed correctly with the *η* about 0.07 for the Pauli measurement set and about 0.2 for the Tetrahedron Platonic Solid measurement set, implying that it requires at least 287 and 820 measurement settings respectively for these two measurement sets to achieve 95% accuracy.

It can be observed from Fig. 2 that: 1)With the increase of measurement rate, the normalized estimation error is reduced, that is, more measurement settings can achieve better estimation accuracy, which is consistent with the Compressive Sensing theory. 2) From the solid lines representing FP_ADMM, even with outliers, the algorithm can still reconstruct the density matrix almost exactly, which corroborates the robustness of FP_ADMM algorithm. 3) For the same number of qubits, FP_ADMM algorithm compared to ADMM or LS algorithm significantly reduces the estimation error. For example, when *n* = 5, and the measurement rate is 0.2, the normalized estimation error of ADMM algorithm is 0.5981, that is, the reconstruction accuracy is 40.19%. The error of LS is 0.8109, and the accuracy of LS is 18.91%. The normalized estimation error of FP_ADMM algorithm is 0.004 under the same measurement rate, and the reconstruction accuracy is 99.60%. Reconstruction accuracy increased by 147.82% compared to ADMM and 426.7% compared to LS. Clearly, the FP_ADMM algorithm significantly improves the reconstruction accuracy of the quantum state estimation via CS.

It can be observed from Table 1 that: 1) For the quantum system having the same number of qubits, with the increase of the measurement rate, the time of the two algorithms’ single iteration goes up. This is because, with the increase in the measurement rate *η*, the number of measurement settings *M* will increase, and the measurement matrix *A* will become a larger matrix (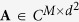), leading to more computation, and the single iteration time increasing. 2) For the same measurement rate, with the increase of the qubit, the single iteration time of the two algorithms increases dramatically. This is due to the increase of qubits leading to an exponential increase in the elements of the density matrix (*d* = 2^*n*^). Even under the same measurement rate, the number of measurement settings grows dramatically, resulting in the increase of the single iteration time. 3) With the same number of qubits and the same measurement rate, the single iteration time of the FP_ADMM algorithm is significantly reduced compared to the ADMM algorithm. With the increase of the qubit, the magnitude of the decrease is increased. When the measurement rate is 0.25, and *n* = 5, for example, the FP_ADMM algorithm is (0.558–0.052) /0.052 = 9.7 times faster than the ADMM algorithm. In addition single iteration the time is 14.3 times faster when *n* = 6, and 155.8 times faster when *n* = 7. It can be concluded that the FP_ADMM algorithm has a higher time efficiency than the ADMM algorithm in the quantum state estimation via CS. With the increase of the system qubit, the time efficiency is more obvious.

## Methods

### Compressive Quantum State Estimation Algorithm using ADMM

Li^26^ is the first to use the ADMM algorithm into quantum state estimation via CS, and achieved better results than the previous algorithms. The ADMM algorithm is an optimization algorithm and has strong robustness. Using the ADMM algorithm to solve (5), the steps in each iteration can be described as follows:

- Fix **S**, *Y*, update *ρ*

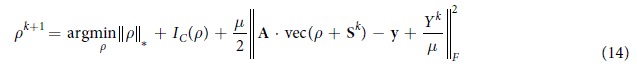
- Fix *ρ*, *Y*, update **S**

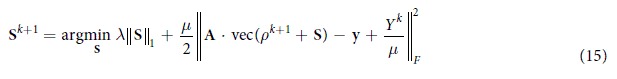
- Fix *ρ*, **S**, update *Y*

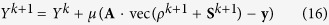


The key of the ADMM algorithm is how to solve (14) and (15). In Li’s algorithm, to solve (14), the process is divided into three steps: the first step is finding a *ρ* that minimizes the Frobenius norm term in (14); the second step, projecting the *ρ* of the first step to a Hermitian matrix; the third step is projecting the result of the second step into a low rank matrix. Similarly, to solve (15), two steps are divided. This involves first minimizing the Frobenius norm term, and then projecting the result of the first step into a sparse matrix. However, in the first step, to minimize the Frobenius norm term, it is necessary to compute the pseudo inverse of a matrix with size *d*^2^ × *d*^2^, which is a large amount of computation.

### ADMM algorithm based on fixed point equation

The fixed point equation^28^ was proposed by Combettes *et al*. in 2005 which can be used to solve optimization problem with certain characteristics. The fixed point equation can describe the solution of an optimization problem as an implicit equation, which is the reason it called fixed point equation.

For an optimization problem with the objective function is 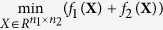, if 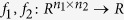 are two proper lower semi-continuous convex functions, and *f*_2_ is differentiable with a 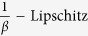 continuous gradient for some *β* > 0, then the solution of the optimization problem satisfies an implicit equation, which is called fixed point equation^15^:


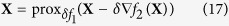


where *δ* ∈ [0, + ∞], and 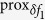 represents the proximity operator of *δf*_1_. The definition of proximity operator of a convex function *ϕ* is 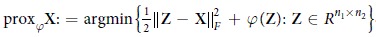. In this paper, we need to use the proximity operator of the nuclear norm and (1, 1) norm^37^,^38^:


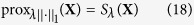



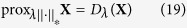


where *S*_*λ*_(*X*) is the soft threshold defined as 
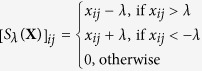
, *D*_*λ*_(**X**) is the singular value contraction operator defined as *D*_*λ*_(**X**) = *US*_*λ*_(*S*)*V*^*T*^, where *USV*^*T*^ is the singular value decomposition of **X**.

Using the fixed point equation (17) to solve the problem (14), it can be divided into two steps:

- First step, find the solution of 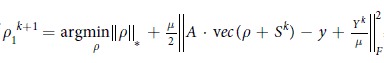, set 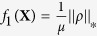, 
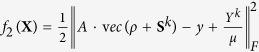
, it can be derived from (17) and (19):
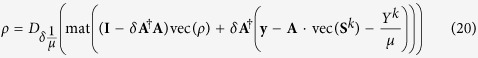
where mat (·) is the operator to convert a vector to a matrix. Deriving from (20), for *ρ* satisfies the implicit equation, the 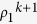 can be solved by iterations as:
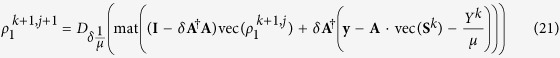
- Second step, project 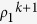 to a Hermitian matrix, and the result denoted as 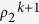. Here we use the method from^26^:

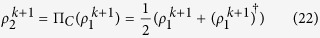


Using the fixed point equation (17) to solve the problem (15), let 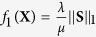, 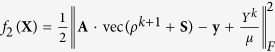, it can be derived from (17) and (18):


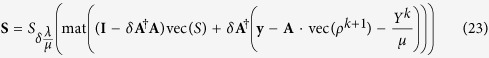


**S** satisfies the implicit equation (23), **S**^*k*+1^ can be updated as follow:


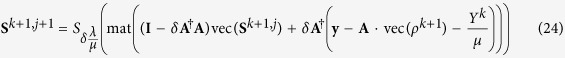


In the iterative procedures to get *ρ*^*k*^, *S*^*k*^ in (21) and (24), a lot of iterations are needed, and the computation takes a lot of time. Inspired by Lin^14^ *et al*.‘s work, we do not have to solve *ρ*^*k*^, *S*^*k*^ exactly. Rather, it turns out updating *ρ*^*k*^, **S**^*k*^ once is sufficient to converge to the optimal solution of problem (14) and (15), which derives the FP_ADMM algorithm. The details are described as follow:


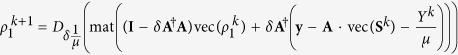



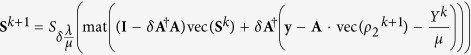



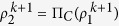



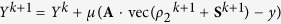


In (14), the use of the fixed point equation can solve 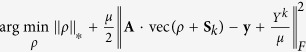 directly. Then, projecting it into a Hermitian matrix can simplify the calculation to two steps. In (15), the fixed point equation can solve it directly which reduces it to one step. Moreover, using the fixed point equation to solve (14) and (15) does not require computing the pseudo inverse of matrices, which can significantly reduce the computational complexity, so that the time efficiency of the algorithm is greatly improved. At the same time, by using the fixed point equation we can solve (14) as a whole optimal solution for the first and third terms, and find (15) the overall optimal solution. So compared with Li’s ADMM algorithm which solves each term of (14) and (15) separately, the reconstruction results of FP_ADMM algorithm achieve higher accuracy.

In the procedure of FP_ADMM algorithm, the selection of the parameters is very important. For the simulation in this paper, we set the compromise factor *λ* as
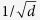
39, and 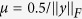. For the algorithm, the stopping criterion is 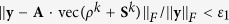 or 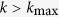, where e_1_ = 10^−7^ and 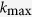 is the maximum number of iterations. For the initialization of FP_ADMM, the initial values of *ρ*, *S* and *Y* are taken as zero matrix, and set *δ* = 1, and *r* = 1.

### Error Analysis

It has been proved that if measurement rate of the partial Pauli measurement set 
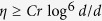
, the measurement matrix satisfies RIP with probability approaching 1, and if 
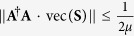
, then the following holds with the same probability^14^:


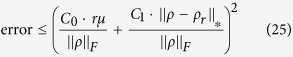


where *C*_0_ and *C*_1_ are positive constant factors. *ρ*_*r*_ is the matrix of rank r that best approximates *ρ* in the nuclear norm. That is, *ρ*_*r*_ is the optimal solution of the problem: 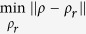 s.t. *rank* (*ρ*_*r*_) ≤ *r*. There are two items in the brackets of (25), the first item depends on the noise *S*, and the second item is the rank-*r* approximation error. However, when 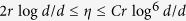 corresponding to (11) (here we set *β* = 1), the the normalized error described in (25) is very difficult to analyze. In quantum state estimation via CS by using Pauli measurement set, the dual certification theory is usually used to explore the lower bound of the number of measurement settings, and the rank RIP theory is used to analyze the upper bound of error.

## Additional Information

**How to cite this article:** Zheng, K. *et al*. A reconstruction algorithm for compressive quantum tomography using various measurement sets. *Sci. Rep.* **6**, 38497; doi: 10.1038/srep38497 (2016).

**Publisher's note:** Springer Nature remains neutral with regard to jurisdictional claims in published maps and institutional affiliations.

## Figures and Tables

**Table 1:** The Comparison of single iteration time between FP_ADMM and ADMM.

*n*	5			6			7		
*η*	0.1	0.25	0.4	0.1	0.25	0.4	0.1	0.25	0.4
FP_ADMM	0.046	0.052	0.058	0.666	0.845	1.018	1.825	1.924	2.241
ADMM	0.500	0.558	0.637	10.59	12.89	15.134	255.831	301.592	326.936

All Timing were performed in MATLAB on a computer with 2 cores of 2.4 GHz Intel Xeon E5-2407 CPUs.
